# Follicular dendritic cell sarcoma aggravated by hyaline-vascular Castleman's disease in association with paraneoplastic pemphigus: study of the tumor and successful treatment^☆^^☆☆^

**DOI:** 10.1016/j.abd.2019.09.009

**Published:** 2019-09-30

**Authors:** Jing Wang, Xiaoyue Wang, Juan Xu, Pengfei Song

**Affiliations:** a Department of Dermatology, The Eighth Affiliated Hospital of Sun Yat-sen University, Shenzhen, Guangdong, China; b College of Biological Science, University of California, Davis, USA

**Keywords:** Castleman's disease, Dendritic cell sarcoma, follicular, Pemphigus

## Abstract

The authors have successfully treated and monitored a case of paraneoplastic pemphigus in association with follicular dendritic cell sarcoma aggravated by hyaline-vascular Castleman's disease. The patient was a 56-year-old female who presented with recalcitrant erosive lichen planus of the oral cavity, tongue, and genital mucosa, along with polymorphous eruptions throughout her body. Histological examination of the cutaneous lesions, indirect immunofluorescence on rat bladder epithelium, and western blot of human keratinocyte proteins identified anti-epidermal antibodies in the patient's serum. Positron emission tomography and computed tomography scans found a mass in her retroperitoneal region. Pathology and immunohistochemistry investigation further corroborated the diagnosis of follicular dendritic cell sarcoma originated from hyaline-vascular Castleman's disease. Complete remission was achieved and the patient has been monitored for four years.

## Introduction

Paraneoplastic pemphigus (PNP) is an autoimmune bullous disease characterized by complications with specific neoplasms. Castleman's disease is the most commonly reported malignancy associated to PNP in China. Follicular dendritic cell sarcomas (FDCS) are relatively rare tumors, which are very closely related to immune reactions and auto-immune diseases. A case of PNP in association with follicular dendritic cell sarcoma that apparently originated from Castleman's disease was studied and successfully treated. The research was not funded by any grants.

## Case report

**Figure 1:**
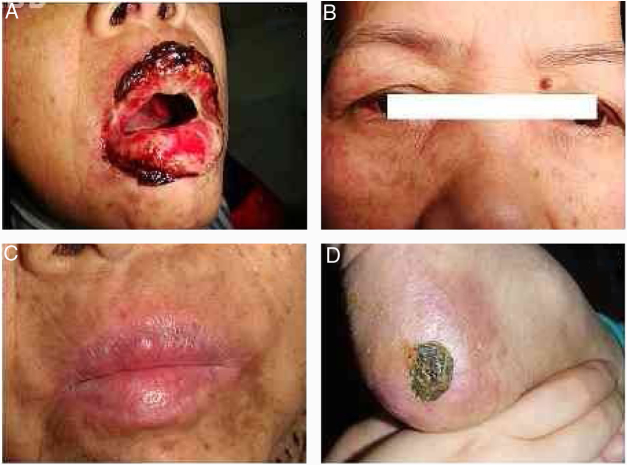
Patient's clinical manifestations. (A) Dermatological examination revealed typical hemorrhagic crusting ulcers on the lips, as well as white-to-yellow exudates and erosions on the lateral surface of tongue; (B) red papules on the face and eyelids, and conjunctiva congestion and erosion in the right eye; (C) skin lesions healed in two months and oral ulcers in six months after removal of the associated follicular dendritic cell sarcomas. No recurrent mucocutaneous lesions were found in four years; (D**)** Hyperkeratosis on her metatarsus large ulcer with hemorrhagic crust on the heel.

**Figure 2:**
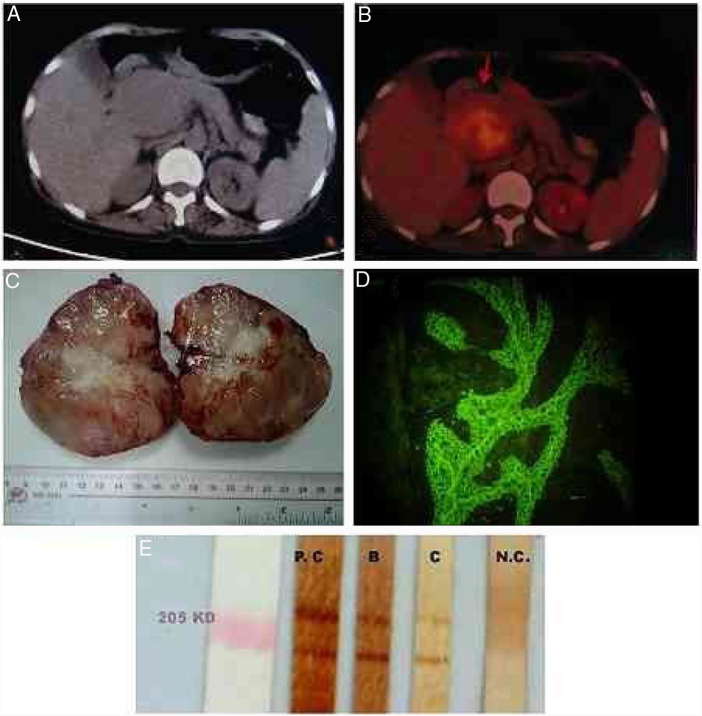
Image exams, macroscopical observation of the tumor, and identification of autoantibodies in patient's serum. (A) Computed tomography (CT) scan showing solitary masses of approximately 10 × 10 × 8 cm in the retroperitoneal region of abdomen; (B) positron emission tomography/CT showing a hypermetabolic mass behind the head of the pancreas suggesting the presence of a malignant mesenchymal tissue tumor; (C) the 10 × 8 × 8 cm solitary masses removed from the patient. Cross sections showed gray-to-yellow color, with fleshy appearance; (D) indirect immunofluorescence of patient's serum showed IgG deposition in intercellular spaces of rat bladder epithelium; (E) Western blot, the serum recognized the 190 kDa periplakin and 210 kDa envoplakin bands of human keratinocyte proteins. Stripe B was from the present case, stripe C was from another paraneoplastic pemphigus patient; PC, positive control; NC, negative control.

The patient was a 56-year-old female who had had oral and lip ulcers for one month and a rash for ten days. The dermatological examination found widespread erosions and shallow ulcers in her oral cavity, tongue, lips, conjunctiva, and genital and anal areas (Fig. 1A and B). Polymorphous eruptions including erythema, vesicles, angry red papules, and plaques were observed on her trunk and distal extremities. She also presented with hair loss, and papules and scars on her scalp. She had dyspnea and dry cough for two weeks. Pulmonary function test showed mixed-type ventilatory defect. Chest X-ray and computed tomography (CT) showed collapse of the bilateral middle and lower lobes of the lungs with ground glass opacity, which suggested bronchiolitis obliterans. A solitary mass of 10 × 8 × 8 cm in size was found in her abdomen by ultrasonic examination; CT and positron emission tomography (PET) scans showed a hypermetabolic mass behind the head of the pancreas (Fig. 2).

Indirect immunofluorescence revealed the deposition of IgG in the intercellular spaces of rat bladder epithelium. Western blot demonstrated anti-epidermal antibodies in patient serum that recognized the antigens of envoplakin, periplakin, desmoglein 3, and linker regions of plakin-family proteins (Fig. 2).^1^ ELISA results for antidesmoglein 3 antibody (MBL – Japan) were 108 (positive). Direct immunofluorescence from patient skin lesion showed deposition of IgG in the intercellular space of the epidermis.

Histological examination of the skin lesion biopsy demonstrated typical intraepidermal acantholytic blisters, individual keratinocyte necrosis, interface vacuolar degeneration, and diffuse lymphocyte infiltration throughout the dermis.

The histological examination of the removed tumor showed several areas with storiform arrangement of spindle cells, intermingled with some small lymphocytes. The chromatin was vesicular, with small nucleoli and mild to moderate variation in nuclear size. Morphological examination suggested a diagnosis of follicular dendritic cell sarcoma. As the tumors were located in the retroperitoneum and in the presence of many blood vessels in the background along with occasional fibrous bands, an underlying hyaline-vascular Castleman's disease was suggested. However, the typical follicles were rare.

**Figure 3:**
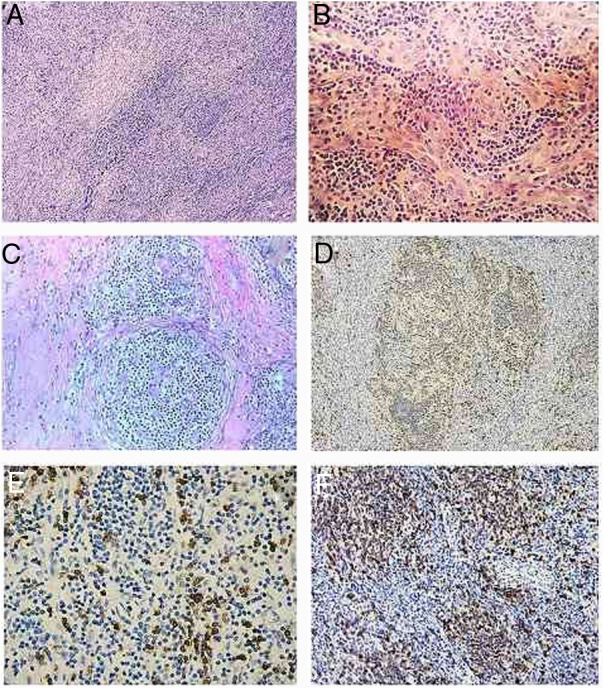
Hematoxylin & eosin and immunochemistry staining of the tumor. (A) Histological examinations showed several areas with storiform arrangement of a large number of spindle or ovoid dendritic tumor cells, intermingled with small lymphocytes and many blood vessels in the background, along with occasional fibrous bands (staining, ×100). (B) Chromatin of the spindly tumor cells is vesicular; small nucleoli are present. There is mild-to-moderate variation in nuclear size (hematoxylin & eosin staining, ×200). (C) Residual follicles observed between the storiform tumor areas with the presence of serpentine and polyvascular follicles typical of cases of hyaline-vascular Castleman's disease. (hematoxylin & eosin staining, ×200). (D) CD23 showed serpentine trabecular meshwork as well as nodular meshwork penetrated by multiple blood vessels (immunochemistry staining, ×100). (E) CD21 positive in regions of the cells bordering the residual follicles; (immunochemistry staining, ×200). (F) CD3 positive, lymphocytes shown in the residual follicles and among the tumor spindle cells (immunochemistry staining, ×200).

CD20 and CD21 showed serpentine trabecular meshwork as well as nodular meshwork penetrated by multiple blood vessels. The storiform areas showed positive for CD35, S-100, vemitin, weakly positive to CD21 and CD68. These follicular dendritic cells are obviously outside the follicles (Fig. 3).

Final diagnosis was follicular dendritic cell sarcoma, aggravated by hyaline-vascular Castleman's disease associated with PNP.

The new criteria established for treatment were followed.^2^, ^3^ Surgical resection of the tumor was performed immediately after confirmed diagnosis. Intravenous immunoglobulin (IVIg) was used before, during, and after the surgical procedure. Methylprednisolone 48 mg was administered daily and the dosage was gradually decreased over the next three years.

Skin lesions improved in two months and oral ulcers in six months. After 12 months, indirect immunofluorescence decreased gradually from 1:128 positive to undetectable. The value of ELISA for antidesmoglein 3 antibody decreased from 108 to negative. However, the patient still presented dyspnea and dry cough. Her second chest X-ray suggested that bronchiolitis obliterans still persisted.^4^, ^5^

## Discussion

The patient's antibody titer decreased to undetectable levels 12 months after treatment and there was no recurrence of PNP or tumors after four years. A previous study reported a 27-year-old female with follicular dendritic cell sarcoma that originated from hyaline-vascular Castleman's disease.^4^ Her treatment results have been monitored for more than ten years. In the present study, it was found that the cultured tumor B cells from the resected tumor have the ability to produce autoantibodies that recognized the antigens in the epidermis (data not shown). It was concluded that when a PNP associated tumor is detected, total resection of the tumor is necessary to achieve complete resolution or improvement of the disease. IVIg should be used before, during, and after the surgical procedure.^2^, ^4^

According to a study on PNP associated tumors, Castleman's disease is the most common lymphoproliferative disorder.^2^ Its histopathological pattern is subdivided into hyaline vascular (HVCD) and plasma cell variants. Infections are the most frequent causes of patient death in these cases. FDCS is a rare tumor, which is commonly misdiagnosed as lymphoma. However, some of the FDCS can originate from Castleman's disease.^5^ In this study, histopathology and immunochemistry studies of the tumors confirmed the diagnosis of FDCS. However, the presence of serpentine and polyvascular follicles suggested that the FDCS originated from HVCD. Interestingly, an analysis of 14 cases of HVCD showed that 11 cases consisted of stroma-rich variants of HVCD. All of the 11 cases were extracted from patients with PNP.^5^, ^6^ The reasons why dendritic cells in Castleman's disease show overgrowth in PNP patients remain uncertain. Further studies focusing on the relationships among the origin of antigen stimulations, dendritic cells, and lymphatic cells may help explain the autoimmune pathogenesis of PNP. Bronchiolitis obliterans is commonly associated with PNP and Castleman's disease, and its treatment continues to be a challenge for PNP patients.^7^, ^8^

## Funding

None declared.

## Author's contribution

Jing Wang: Approval of the final version of the manuscript; conception and planning of the study; elaboration and writing of the manuscript; critical review of the literature; critical review of the manuscript.

Xiaoyue Wang: Elaboration and writing of the manuscript.

Juan Xu: Effective participation in research orientation; critical review of the literature; critical review of the manuscript.

Pengfei Song: Obtaining, analyzing and interpreting the data; approval of the final version of the manuscript.

## Conflicts of interest

The authors declare no conflicts of interest.
